# Antimicrobial responses of peripheral and central nervous system glia against *Staphylococcus aureus*

**DOI:** 10.1038/s41598-021-90252-0

**Published:** 2021-05-21

**Authors:** Indra N. Choudhury, Anu Chacko, Ali Delbaz, Mo Chen, Souptik Basu, James A. St John, Flavia Huygens, Jenny A. K. Ekberg

**Affiliations:** 1 Clem Jones Centre for Neurobiology and Stem Cell Research, Griffith University, Nathan, QLD Australia; 2 Menzies Health Institute Queensland, Griffith University, Southport, QLD 4222 Australia; 3 Griffith Institute for Drug Discovery, Griffith University, Nathan, QLD Australia; 4 Centre for Immunology and Infection Control, School of Biomedical Sciences, Faculty of Health, Queensland University of Technology, Brisbane, QLD Australia

**Keywords:** Neurodegeneration, Bacterial host response, Pathogens

## Abstract

*Staphylococcus aureus* infections of the central nervous system are serious and can be fatal. *S. aureus* is commonly present in the nasal cavity, and after injury to the nasal epithelium it can rapidly invade the brain via the olfactory nerve. The trigeminal nerve constitutes another potential route of brain infection. The glia of these nerves, olfactory ensheathing cells (OECs) and trigeminal nerve Schwann cells (TgSCs), as well as astrocytes populating the glia limitans layer, can phagocytose bacteria. Whilst some glial responses to *S. aureus* have been studied, the specific responses of different glial types are unknown. Here, we compared how primary mouse OECs, TgSCs, astrocytes and microglia responded to *S. aureus*. All glial types internalized the bacteria within phagolysosomes, and *S. aureus*-conjugated BioParticles could be tracked with subtle but significant differences in time-course of phagocytosis between glial types. Live bacteria could be isolated from all glia after 24 h in culture, and microglia, OECs and TgSCs exhibited better protection against intracellular *S. aureus* survival than astrocytes. All glial types responded to the bacteria by cytokine secretion. Overall, OECs secreted the lowest level of cytokines, suggesting that these cells, despite showing strong capacity for phagocytosis, have immunomodulatory functions that can be relevant for neural repair.

## Introduction

Bacterial invasion of the central nervous system (CNS) is relatively rare but can result in significant mortality and morbidity. Pathogen invasion of the CNS can occur via the blood brain barrier (BBB) or blood-cerebrospinal fluid barrier (BCSFB). An alternative route is via the nerves that connect the nasal cavity and the CNS, the olfactory nerve (cranial nerve I) and the intranasal branches of the trigeminal nerve (cranial nerve V), which terminate in the olfactory bulb and brainstem, respectively^1,2^. In the olfactory system, the cell bodies of primary olfactory neurons are localised in the olfactory neuroepithelium at the roof of the nasal cavity. Their dendrites extend directly into the nasal cavity, where odorant detection takes place, whilst their axons project all the way into the olfactory bulb in the brain, where they synapse onto second order neurons. Thus, the primary olfactory nervous system is a one-synapse route from the olfactory neuroepithelium directly to the CNS^3^. The olfactory nerve is unique in that it regenerates throughout life^3,4^; perhaps this nerve has evolved to regenerate continuously because its dendrites are exposed to microbes and toxins in the nasal cavity^5^. Whilst cell bodies of trigeminal neurons are localised in trigeminal ganglia far from the periphery, the distal ends of some trigeminal axons that innervate the nasal cavity are close to the apical surface of the nasal epithelium^6^. Despite the anatomy of the olfactory and trigeminal nerves, they appear to be only rarely affected by fulminating microorganism infection.

The intact nasal epithelium usually provides a barrier preventing invasion against most pathogens, and the epithelium contains strong innate and adaptive immune system components, accompanied by those in the nearby nasal-associated lymphoid tissue^7,8^. The nerves are also protected by their respective glia, which have strong innate immune properties. The glia of the primary olfactory nervous system, olfactory ensheathing cells (OECs), which ensheathe bundles of olfactory axons, exhibit capacity for phagocytosis of cell debris resulting from olfactory neuron turnover^9–11^. Both OECs^12–16^ and the glia of the trigeminal nerve, trigeminal Schwann cells (TgSCs)^16^, can respond to and phagocytose bacteria. OECs and Schwann cells have, however, been shown to respond differently to pathogens and pathogen-associated molecular patterns (PAMPs)^14^. Should microbes invade the olfactory or trigeminal nerves, they also encounter a “third layer of defence” when they reach the glia limitans layer, the demarcation between the PNS and CNS, where astrocytes are present^17^. After encountering bacterial antigens, astrocytes both rapidly participate in acute innate immune responses and prompt an adaptive immune response^18,19^. Microglia are the innate immune cells and resident phagocytes of the brain^20^ and they defend the brain from invading bacteria^21^.

A small number of bacteria are thought able to evade these protection mechanisms and invade the CNS via the olfactory and/or trigeminal nerves, such as *Neisseria meningitidis, Streptococcus pneumoniae, Chlamydia pneumoniae, Listeria monocytogenes, Streptococcus pneumoniae, Nocardia cyriacigeorgica* and *Burkholderia pseudomallei*, as well as some viruses, such as herpes simplex type 1 (HSV1)^2,22^ and potentially SARS-CoV-2^23^. Injury to the nasal epithelium may increase pathogen invasion of the underlying nerves, as has been shown for *B. pseudomallei*^24^, and may also allow invasion by microbes present in the nasal cavity, but not normally gaining access to nerves.

One bacterium known to be capable of CNS infection that is commonly present in the nasal cavity is *Staphylococcus aureus*; ~ 50% of healthy adults harbour *S. aureus* in the nasal cavity either persistently or intermittently^25^. *S. aureus* causes 1–9% of bacterial meningitis cases in adults^26,27^ and is one of the most common causes of brain abscess^28,29^. *S. aureus* can infect human microvascular endothelial cells and thus has capacity for crossing the blood–brain barrier (BBB)^30^, but is also thought to use other infection routes^2^. After experimental injury to the nasal epithelium, *S. aureus* can rapidly invade the olfactory bulb via the olfactory nerve^12,13^; thus, this bacterium, once it reaches the olfactory (and potentially the trigeminal nerve) is able to withstand the immune response mounted by peripheral nerve glia.

Some key responses of OECs to *S. aureus* have been studied. Following challenge with *S. aureus*, expression of inducible nitric oxide synthase (iNOS) mRNA was shown to be strongly upregulated in OECs, resulting in nitric oxide (NO) production (measured as nitrite concentration), accompanied by nuclear translocation of nuclear factor kappa B (NFκB). OECs were also shown to express increased amounts of mRNA for key innate immune components, such as lysozyme, interleukin 6 (IL-6) and the chemokine C-X-C motif ligand 1 (CXCL-1; also known as Gro1 or KC) in response to *S. aureus*^12,13^. However, key aspects of OEC responses to *S. aureus* remain to be characterised, such as the time-course of internalization as well as a broad analysis of cytokine and chemokine production. Furthermore, OECs have been shown to mount a much more powerful immune response to *Escherichia coli* and PAMPs than Schwann cells^14^, suggesting differences between types of glia in their handling of pathogens. To date, the responses by different glial types to *S. aureus* have not been compared. Determining how glia in the olfactory and trigeminal nerves, and in the glia limitans layer, respond to different bacteria is the key to understanding how certain pathogens can invade the CNS via peripheral nerves. In the current study, we compared how OECs, TgSCs, astrocytes and microglia responded to *S. aureus* infection, in particular the time-course of internalization and production of a range of cytokines and chemokines.

## Results

### Intracellular survival of *S. aureus* differs between glial types

**Figure 1:**
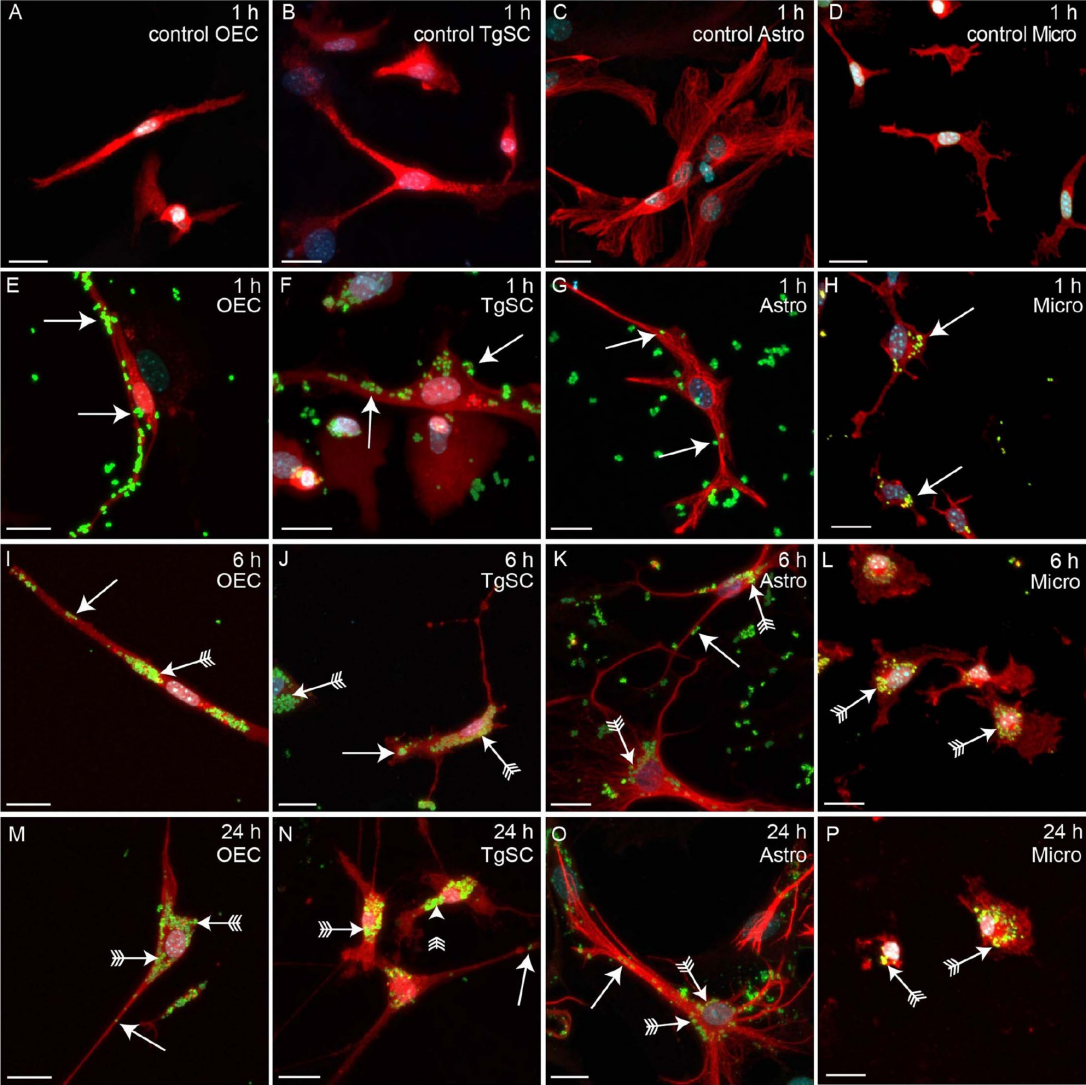
*S. aureus* attaching to and being internalised into OECs, TgSCs, astrocytes and microglia. Panels show primary cultures of OECs (left panels), TgSCs (second panels), astrocytes (third panels) and microglia (right panels) from S100β-DsRed mice, in which all glia express the DsRed protein. For OECs and TgSCs, red labelling shows DsRed; for astrocytes, red labelling shows GFAP (these cells express low levels of DsRed); for microglia, red labelling shows Iba-1 (these cells express low level of DsRed) and Hoechst for nucleus staining. (**A**–**D**) Control wells of the four glial types 1 h post addition of medium without bacteria and antibiotics. (**E**–**H**) At 1 h post exposure to *S. aureus*, bacteria had adhered to the cell surface (arrow) and internalized (arrow) within the microglia. (**I**–**P**) After 1 h, antibiotics were added, preventing survival of bacteria in the medium. (**I**–**L**) *S. aureus* in glia at 6 h post exposure, some bacteria were present in the processes (arrows) while larger amounts accumulated in the perinuclear region (arrows with tail). (**M**–**P**) At 24 h post exposure, most bacteria were present in the perinuclear region (arrows with tail) while some bacteria were also present in the processes (arrows). Scale bar: 20 μm.

**Figure 2:**
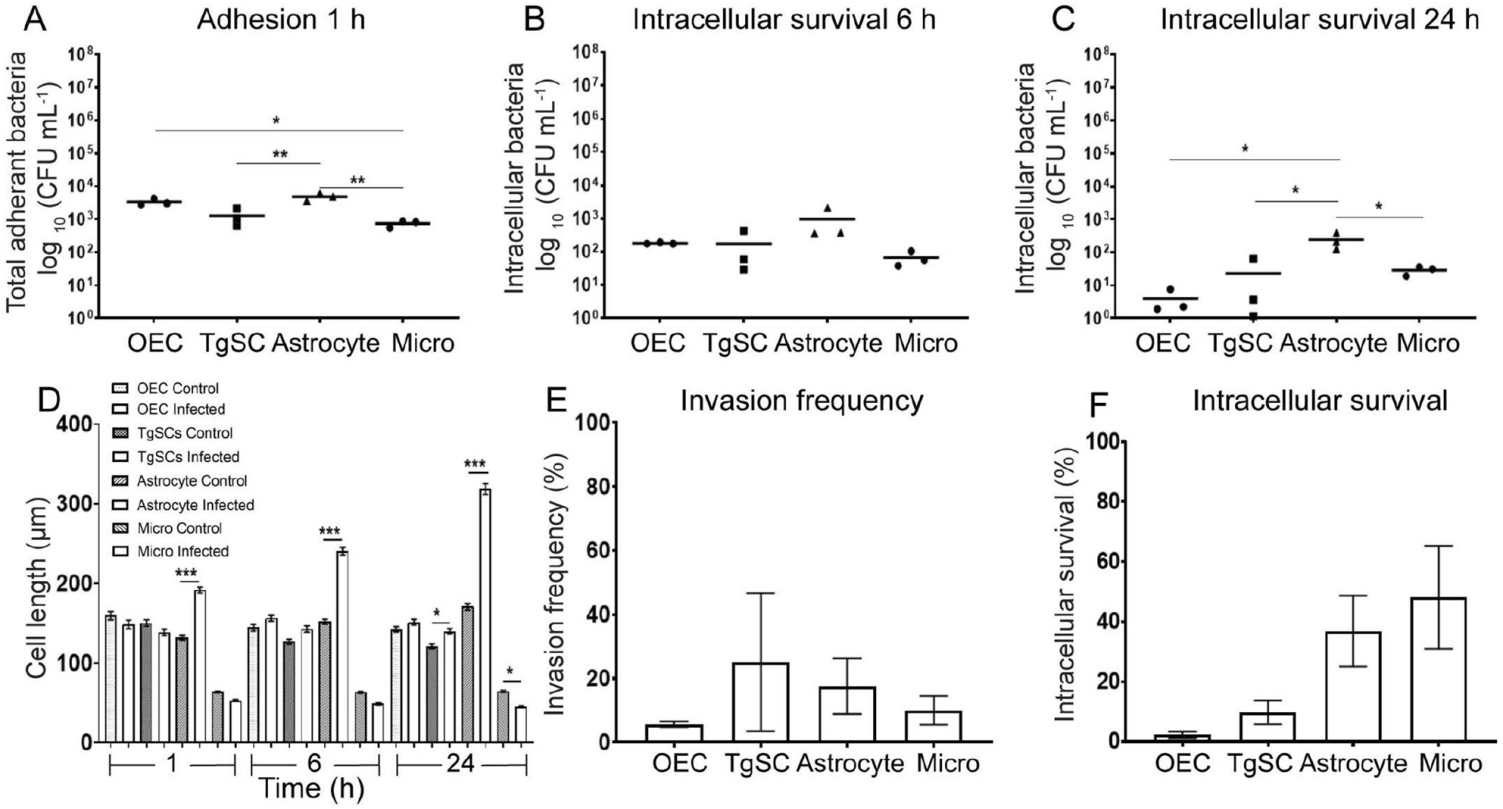
*S. aureus* adhesion to, and invasion of, primary glia*.* (**A**) Adhesion assays. Cell monolayers were incubated with *S. aureus* (MOI 100:1) for 1 h, followed by washing and colony counting to determine the number of bacteria (CFU) that adhered to cells. (**B**,**C**) Intracellular survival assay. After 1 h of exposure to *S. aureus*, a combination of three antibiotics was added to the medium to remove extracellular bacteria. After 6 h (**B**) or 24 h (**C**), cells were lysed to recover intracellular bacteria, and colony counts were performed. Results (**A**–**C**) were normalized with the number of viable glia cells. (**D**) Cell morphology (length) measurement. Manual measurement of cell length (10–15 cells/Field of view (FOV) with 10–15 FOV, a total of 200 cells were counted manually) using NIS software at different time-points after exposure to *S. aureus* with control wells. (**E**) Invasion frequency. The percentages of initially adhered bacteria that later invaded the cells was determined (CFUs isolated from cells at 6 h [B] divided by CFUs adhering to cells at 1 h [A]). (**F**) Intracellular survival. The percentage of bacteria inside cells was compared between the 24 h and 6 h time-point (CFUs isolated from cells at 24 h divided by CFUs isolated from cells at 6 h). Results for (**E**,**F**) were normalized with the number of viable glia cells. For (**A**–**D**) data shows mean ± SEM (two way ANOVA with Tukey’s multiple comparison test), (**E**–**F**) bars show mean ± SEM, (Kruskal–Wallis test with Dunn’s multiple comparison), n = 3 biological and 3 technical replicates (3 wells with 4000 cells per well), *p ≤ 0.05, **p ≤ 0.01, ***p ≤ 0.001.

To investigate the susceptibility of different glial cell types to *S. aureus* infection, we compared the adhesion and invasion capacity of live *S. aureus* between primary OECs and TgSCs (PNS glia from the olfactory and trigeminal nerve, respectively) with astrocytes and microglia (CNS glia). We first exposed the cells to *S. aureus* for 1 h, and then applied antibiotics to the medium to inhibit extracellular survival of the bacteria after which cells/bacteria were incubated for a further 6 h and 24 h. At 1 h after commencement of the assay, OECs and TgSCs that were not inoculated with *S. aureus* exhibited typical bipolar morphology, while astrocytes had multi-branched broad morphology and microglia showed a resting or ramified morphology (Fig. 1A–D). When the cells were inoculated with *S. aureus*, bacteria were present along the length of the processes of the OECs and TgSCs (Fig. 1E–F). While there appeared to be minor changes to the cell morphology, with some cells becoming broader while other became more elongated. Measurements of cell length revealed that the presence of *S. aureus* did not alter overall cell length of OECs, TgSCs and microglia at 1 h and 6 h (Fig. 2D). At 24 h, however, TgSCs exposed to *S. aureus* were significantly longer than control cells (Fig. 2D). In contrast, astrocytes underwent considerable morphological changes when exposed to the bacteria; their overall lengths were significantly greater at all timepoints (Figs. 1G, 2D). Microglia showed uptake of bacteria localised in the perinuclear area at 1 h (Fig. 1H). At 24 h though a significant decrease in size of infected microglia was observed (Fig. 2D). After the antibiotic protection media was added to prevent extracellular survival of the bacteria, the bacteria were clearly internalised within the cells at 6 h post exposure for all glial cells. Some bacteria were present in the processes of the cells (arrows, Fig. 1I–K), but majority of bacteria appeared to be localised in the perinuclear region (arrows with tails). At 24 h, the bacteria were mainly in the perinuclear region of OECs, TgSCs and microglia (Fig. 1M–N) with few bacteria localised in the processes of OECs, TgSCs and microglia (Fig. 1M–N,P). In contrast, while bacteria were in the perinuclear region of astrocytes, there appeared to be more bacteria present along the branches (arrows, Fig. 1O).

To quantify the amount of bacteria that adhered or were internalised into cells, cells were washed and then lysed at different times and lysates were plated onto selective BHI agar plates. We observed significant differences between types of glia regarding *S. aureus* adherence to the cells. Significantly more bacteria adhered to astrocytes than to TgSCs and microglia after 1 h (Fig. 2A). OECs showed significantly higher bacterial adherence than microglia too (Fig. 2A). Following antibiotic treatment at 1 h to prevent extracellular survival of bacteria, and subsequent analysis at 6 h post exposure to *S. aureus*, live bacteria could be isolated from all three glial types. There was, however, no difference in the amount of bacteria between the glial types (Fig. 2B). In contrast, at 24 h post exposure, there were significantly more bacteria in astrocytes as compared to OECs, TgSCs and microglia, suggesting a decrease in intracellular survival or a more efficient bacterial killing by OECs, TgSCs and microglia as compared to astrocytes (Fig. 2C). We analysed the invasion frequency (the percentage of the initially adhered bacteria that were inside cells at 6 h); there was no significant difference in invasion frequency between cell types (Fig. 2E). We also determined the percentage of bacteria that survived intracellularly (amount of bacteria isolated from inside cells at 24 h compared to 6 h) and found no significant difference (Fig. 2F). To account for changes in cell numbers during the assay (proliferation or death), a cell count was performed and intracellular survival was calculated on the cell numbers at 24 h (OECs, TgSCs and astrocytes showed no significant change in cell numbers; only microglia showed a significant decrease in cell number over the assay). Thus the effect observed in the results is due to glial responses to bacteria and not due to cell death occurring post infection.

### Phagocytosis of *S. aureus* and pHrodo *S. aureus* BioParticles by glial cell types

**Figure 3:**
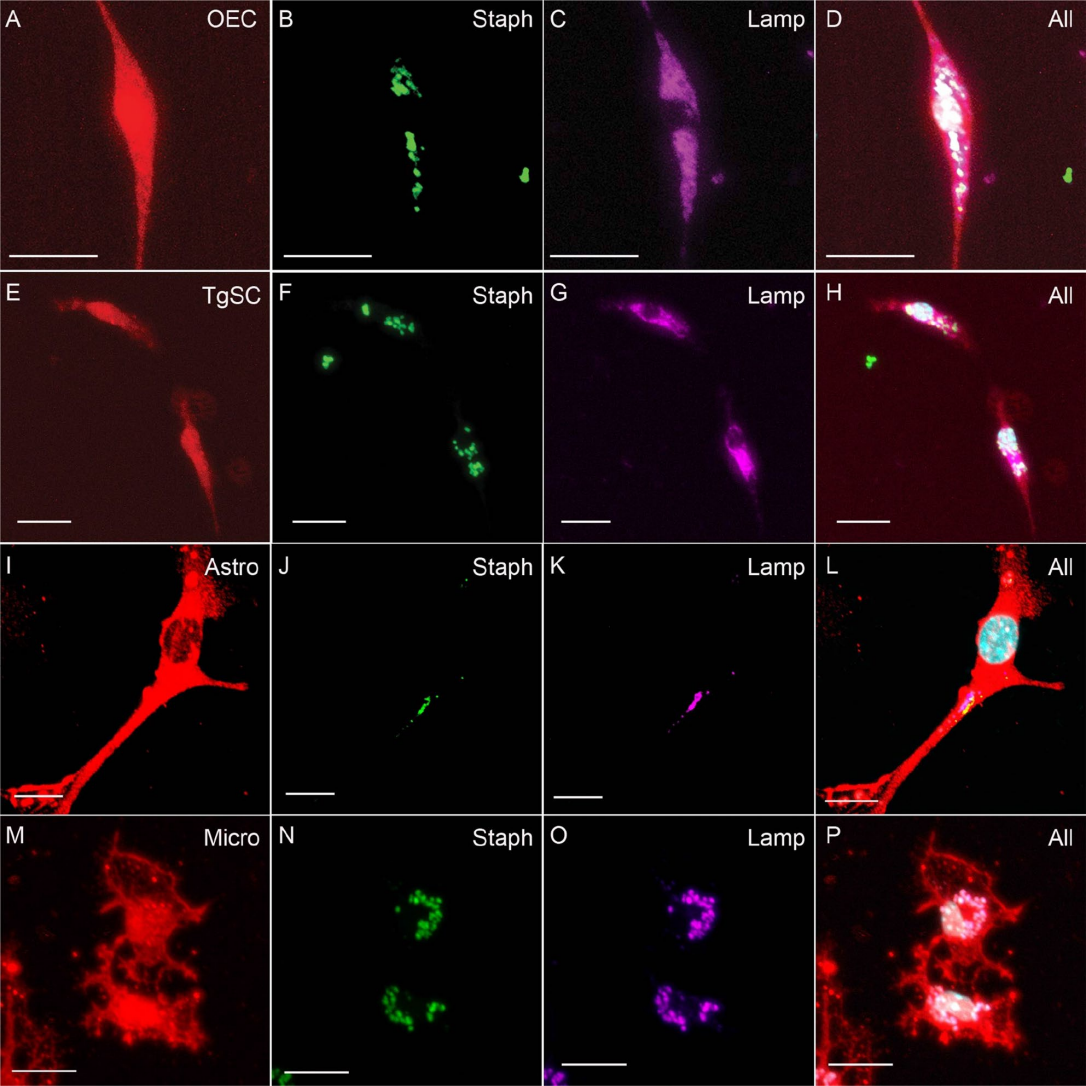
Phagocytosis of *S. aureus* in phagolysosomes in different glial cells. Panels show primary cultures of OECs (**A**–**D**), TgSCs (**E**–**H**), astrocytes (**I**–**L**) and microglia (**M**–**P**) from S100β-DsRed mice. For OECs and TgSCs, red labelling shows DsRed; for astrocytes, red labelling shows GFAP (these cells express low levels of DsRed); for microglia, red labelling shows Iba-1 and Hoechst for nucleus staining. (**B**,**F**,**J**,**N**) shows *S. aureus* within cells, (**C**,**G**,**K**,**O**) LAMP-2 stain for lysosomes. *S. aureus* colocalizes with LAMP-2 stains showing the bacteria is internalized in phagolysosomes. Scale bar: 20 μm.

To determine whether the glia phagocytosed *S. aureus* and internalised the bacteria in lysosomes, we stained the cells with a lysosomal membrane protein 2 marker (LAMP-2) which is a protein component of the lysosome membrane. The lysosomes function to fuse to foreign particles to form a phagolysosome which then aids in their acidification and degradation. We observed that all glial cells showed positive LAMP-2 staining around the bacterial stains of *S. aureus* (Fig. 3) demonstrating that the bacteria was internalized or colocalized inside lysosomes.

**Figure 4:**
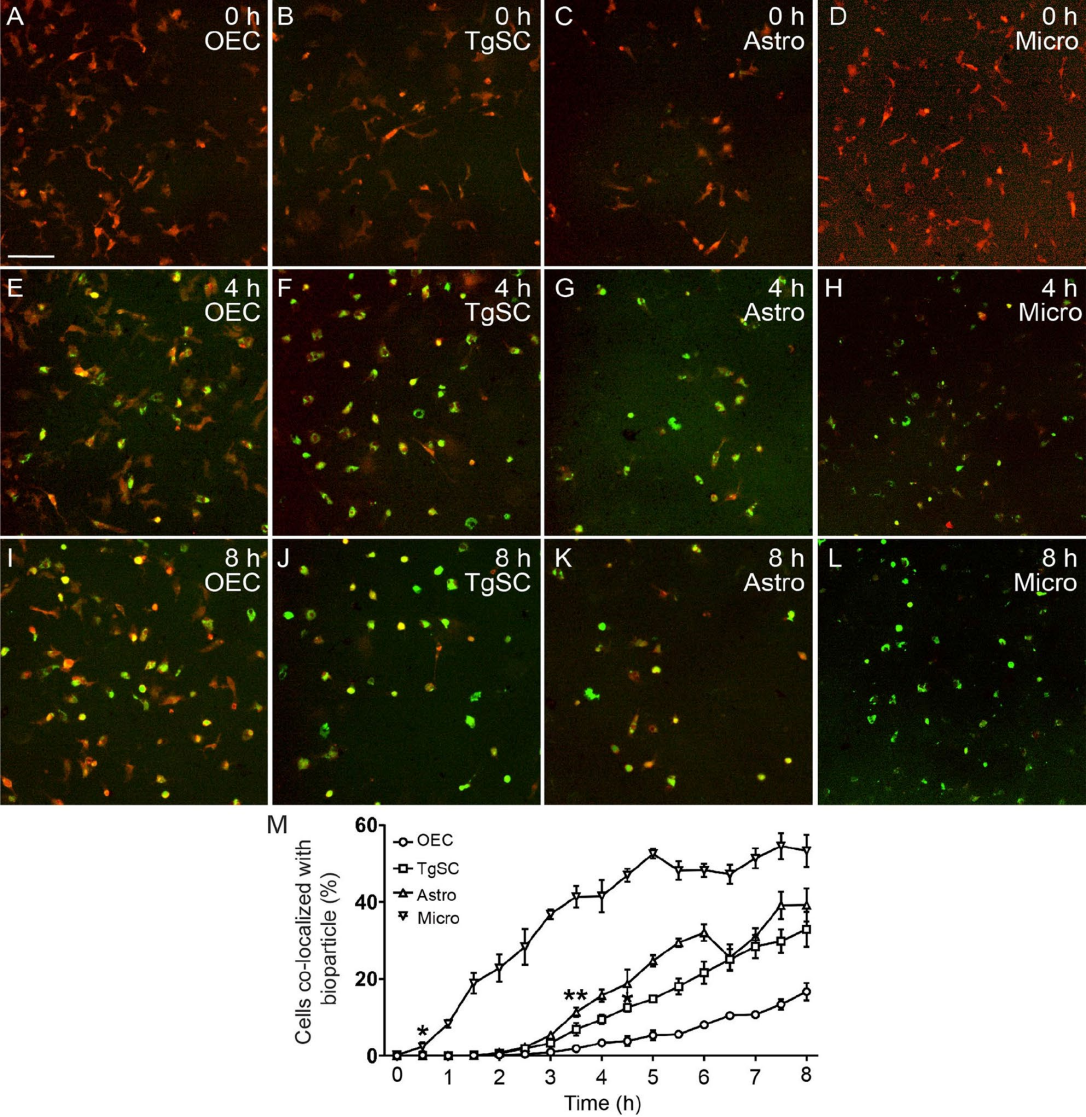
Phagocytosis of pHrodo *S. aureus* BioParticles by different glial cell types. (**A**–**L**) Images show fluorescent *S. aureus* BioParticles (which exhibit green fluorescence when inside lysosomes) co-localized with the glia (red; primary cultures from S100β-DsRed mice) at 4 h and 8 h post exposure. Since this is a live-cell assay, astrocytes (Astro) and microglia (micro) could not be immunolabeled and had to be identified based on their (weak) expression of DsRed. (**M**) Time-course of phagocytosis assay showing the percentage of glia that contained phagocytosed fluorescent *S. aureus* BioParticles at different time-points. Significant difference was observed at 30 min between micro and other glial cells, 3.5 h between Astro and OEC, at 4.5 h between TgSC and OEC. Data show mean ± SEM, *p ≤ 0.05, **p ≤ 0.01, two-way ANOVA with Tukey’s multiple comparison test, n = 3 biological replicates × 4000 cells per well. Scale bar: 100 μm.

To determine the time course of glial phagocytosis of *S. aureus* (resulting in the bacteria being internalized into lysosomes) we exposed the glia to pH-sensitive (pHrodo) *S. aureus* BioParticles, which exhibit fluorescence only when in an acidic environment (i.e. in phagolysosomes). The phagocytosis assay was performed using the four types of glia for increasing durations ranging from 30 min to 8 h, with imaging of the cells every 30 min (Fig. 4). The cells internalized an increasing amount of BioParticles over time (Fig. 4M). In microglia, a significantly higher proportion of cells internalized the BioParticles at 30 min, compared to astrocytes, TgSCs or OECs. Significantly more astrocytes and TgSCs internalised the BioParticles than OECs. BioParticle internalization into acidic cellular compartment differed between astrocytes and OECs after 3.5 h (Fig. 4M). At 4.5 h post exposure, TgSCs contained more phagocytosed particles than OECs (Fig. 4M) .

### Glia produce multiple cytokines and chemokines after exposure to *S. aureus*

**Figure 5:**
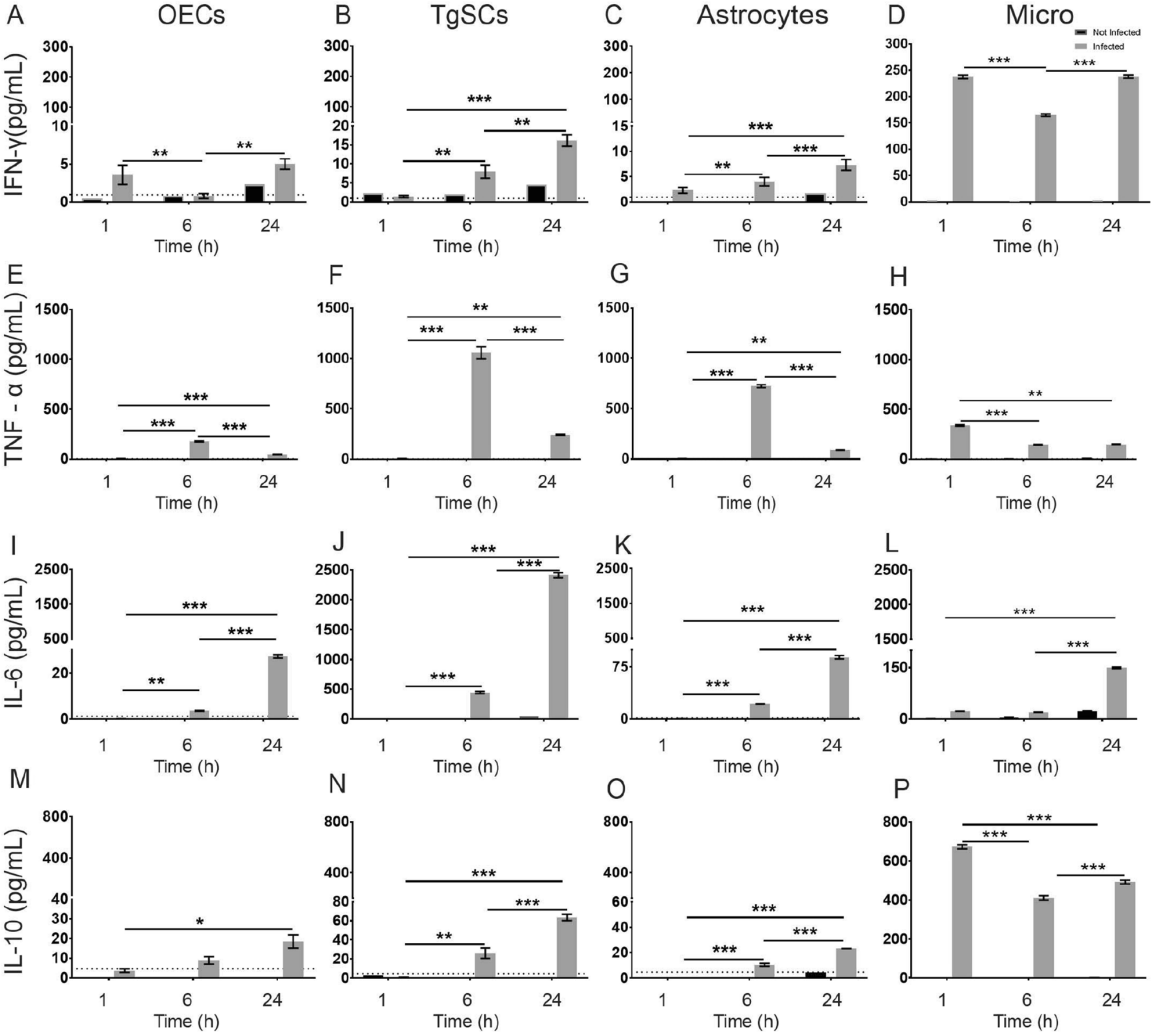
Cytokine responses in glia post *S. aureus* infection. Cytokine production by OECs, TgSCs, astrocytes and microglia were measured using multiplex ELISA at 1 h, 6 h and 24 h post exposure to *S. aureus*. The graphs show the amount of the pro-inflammatory cytokines IFN-γ (**A**–**D**), TNF-α (**E**–**H**), and IL-6 (**I**–**L**) and regulatory cytokine IL-10 (**M**–**P**). Data shows mean ± SEM. (*p ≤ 0.05, **p ≤ 0.01, ***p ≤ 0.001 (two-way ANOVA with Tukey’s multiple comparison test), n = 2 technical replicates (100,000 cells/well).

**Figure 6:**
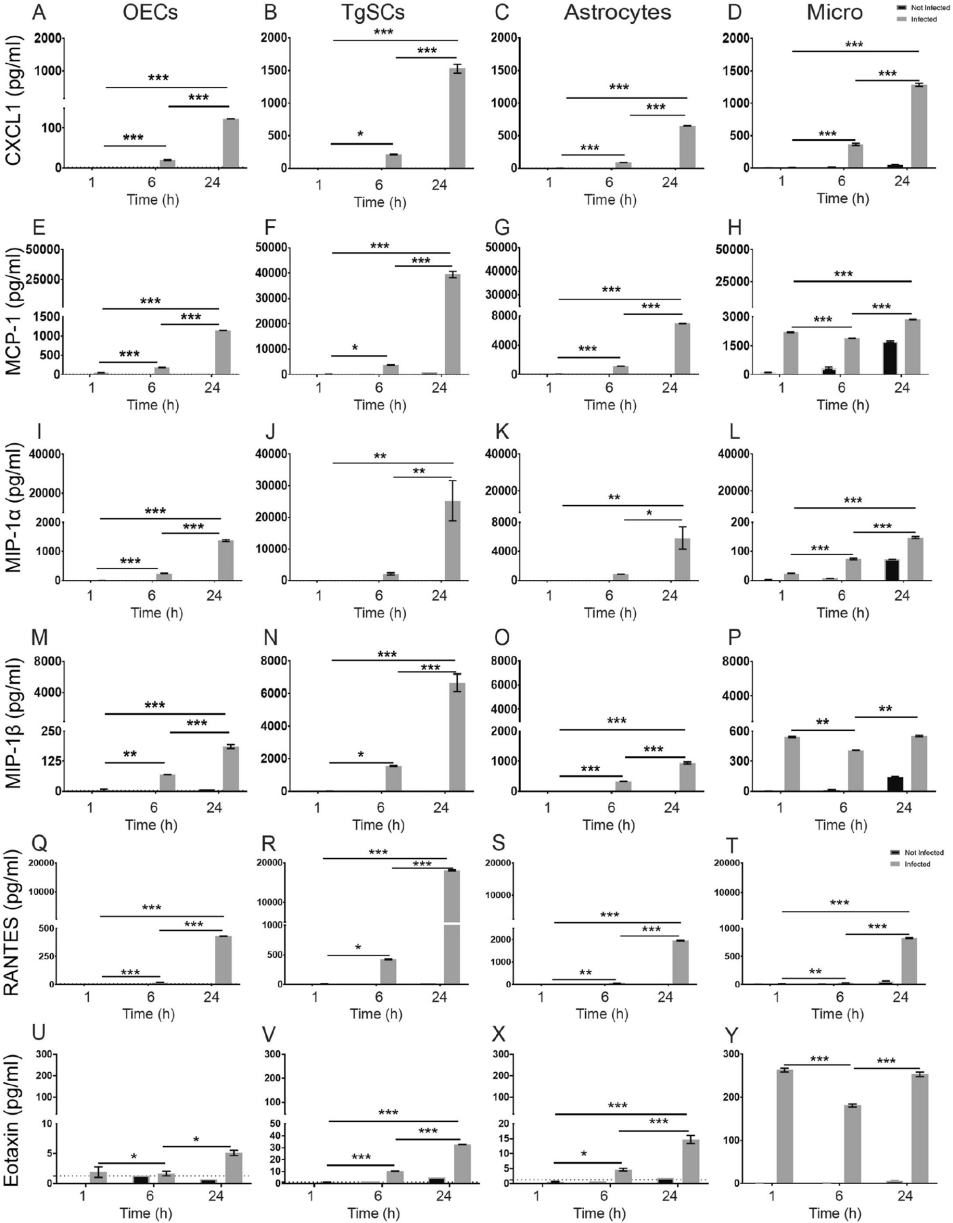
Production of chemokines by the different glial types following exposure to *S. aureus*. Chemokine production by OECs, TgSCs, astrocytes and microglia were measured using multiplex ELISA at 1 h, 6 h and 24 h post exposure to *S. aureus*. CXCL-1(**A**–**D**), MCP-1 (**E**–**H**), MIP-1α (**I**–**L**), MIP-1β (**M**–**P**), RANTES (**Q**–**T**) and Eotaxin (**U**–**Y**). Data shows mean ± SEM. *p < 0.05, **p < 0.01, ***p < 0.001 (two-way ANOVA with Tukey’s multiple comparison test), n = 2 technical replicates (100,000 cells/well).

To gain insight into the innate immune responses to *S. aureus* by the different glia, we measured the production of cytokines and chemokines at different time-points post exposure to bacteria (1, 6 and 24 h post exposure). The results showed significant production of several cytokines (Fig. 5) and chemokines (Fig. 6) as early as 1 h post exposure to *S. aureus* by all four glia types. The response included increased secretion of pro-inflammatory cytokines interferon γ (IFN-γ) and tumour necrosis factor α (TNF-α), with levels of IFN-γ being high in microglia than TNF-α and lower than those for OECs; TgSCs and astrocytes (Fig. 5A–H). The cells also responded to *S. aureus* with secretion of interleukin 6 (IL-6), and the anti-inflammatory and immune-regulatory cytokine interleukin 10 (IL-10). The amount of IL-6 was consistently higher than the amount of IL-10 (Fig. 5) in OECs, TgSCs and astrocytes; in contrast microglia expressed higher IL-10 than IL-6. Microglia produced significantly higher levels of IFN-γ and IL-10, TgSCs showed significantly higher levels of TNF-α and IL-6, than the other glial types, with some variations between time-points (Fig. 5 and Table 1). Overall, production of TNF-α by OECs, TgSCs and astrocytes peaked at 6 h post exposure and for microglia at 1 h. The levels of IFN-γ and IL-10 were highest at 1 h for microglia as compared to OECs, TgSCs and astrocytes and continued till 24 h. Whilst the levels of IFN-γ, IL-6 and IL-10 were highest at 24 h for OECs, TgSCs and astrocytes.

**Table 1:** Cytokine/chemokine secretion by OECs, TgSCs, astrocytes and microglia post *S. aureus* exposure with comparison between the glia.

Cytokine	Time	OEC (pg/mL)	TgSC (pg/mL)	Astro (pg/mL)	Micro (pg/mL)	OEC *vs* TgSC	OEC *vs* Astro	TgSCs *vs* Astro	OEC vs Micro	TgSC vs Micro	Astro vs Micro
IFN-γ	1 h	3.6 ± 1.3	1.4 ± 0.3	2.3 ± 0.6	237.5 ± 3.5	ns	ns	ns	***	***	***
	6 h	0.8 ± 0.3	8.0 ± 1.7	4.0 ± 0.8	164.7 ± 2.4	***	ns	*	***	***	***
	24 h	5.0 ± 0.7	16.2 ± 1.5	7.3 ± 1.1	238.1 ± 3.1	***	ns	***	***	***	***
TNF-α	1 h	5.2 ± 1.1	7.6 ± 1.3	5.2 ± 0.2	336.2 ± 7.1	ns	ns	ns	***	***	***
	6 h	176.8 ± 5.1	1056.7 ± 60.6	722.5 ± 14.3	143 ± 4.2	***	***	***	ns	***	***
	24 h	44.1 ± 0.9	242.8 ± 4.5	88.5 ± 1.1	147 ± 4.2	***	ns	***	***	***	ns
IL-6	1 h	0.1 ± 0.1	6.4 ± 0.2	0.2 ± 0.1	22.2 ± 0.2	ns	ns	ns	ns	ns	ns
	6 h	3.5 ± 0.1	442.8 ± 19.2	21.2 ± 0.2	19.3 ± 0.5	***	ns	***	ns	***	ns
	24 h	27.3 ± 0.7	2412.3 ± 42.5	89.0 ± 2.5	149.4 ± 2.1	***	**	***	***	***	**
IL-10	1 h	3.7 ± 0.9	0.4 ± 0.4	0	673.2 ± 11.0	ns	ns	ns	***	***	***
	6 h	8.9 ± 1.9	26.0 ± 5.4	10.4 ± 1.1	410.4 ± 11.6	***	ns	***	***	***	***
	24 h	18.5 ± 3.4	63.5 ± 3.3	23.1 ± 0.1	492.2 ± 9.7	***	ns	***	***	***	***
CXCL-1	1 h	0	0	0.6 ± 0.6	8.8 ± 0.3	ns	ns	ns	ns	ns	ns
	6 h	20.4 ± 1.2	212.0 ± 8.1	87.9 ± 0.2	368.6 ± 15.9	***	ns	***	***	***	***
	24 h	123.1 ± 0.3	1527.7 ± 69.2	652.0 ± 4.0	1285 ± 21.7	***	***	***	***	***	***
MCP-1	1 h	50.0 ± 4.5	234.1 ± 5.5	52.1 ± 6.6	2197 ± 21.1	ns	ns	ns	***	***	***
	6 h	179.4 ± 7.4	3806.9 ± 150.9	1139.0 ± 13.7	1877 ± 16.2	***	ns	***	**	***	ns
	24 h	1145.8 ± 3.4	39,399.4 ± 1259.5	6940.4 ± 35.6	2865 ± 23.9	***	***	***	**	***	***
MIP-1α	1 h	2.4 ± 0.1	5.4 ± 0.2	3.1 ± 0.1	24.4 ± 0.6	ns	ns	ns	ns	ns	ns
	6 h	240.1 ± 8.9	2083.6 ± 361.3	879.1 ± 31.5	73.4 ± 2.8	ns	ns	ns	ns	ns	ns
	24 h	1376.1 ± 32.3	25,274.2 ± 6374.0	5865.0 ± 1529.2	148.1 ± 3.1	***	ns	***	ns	***	ns
MIP-1β	1 h	4.4 ± 4.4	8.8 ± 0.6	0	541.8 ± 6.1	ns	ns	ns	*	*	*
	6 h	68.8 ± 0.8	1544.2 ± 41.1	333.0 ± 2.2	411.5 ± 1.4	***	ns	***	ns	***	ns
	24 h	186.2 ± 7.9	6663.1 ± 535.5	943.6 ± 4	552.8 ± 8.1	***	**	***	ns	***	ns
RANTES	1 h	0	12.8 ± 0.5	0.3 ± 0.3	12.5 ± 0.2	ns	ns	ns	ns	ns	ns
	6 h	20.6 ± 0.5	425.1 ± 9.0	68.8 ± 0.4	30.4 ± 1.2	***	ns	***	ns	***	ns
	24 h	430.9 ± 1.8	18,114.8 ± 141.7	1959.0 ± 20.9	829.2 ± 7.3	***	***	***	***	***	***
Eotaxin	1 h	1.9 ± 0.9	1.0 ± 0.3	0.6 ± 0.2	263.3 ± 4.5	ns	ns	ns	***	***	***
	6 h	1.7 ± 0.4	10.4 ± 0.2	4.7 ± 0.5	181.1 ± 3.5	***	***	**	***	***	***
	24 h	5.1 ± 0.5	32.8 ± 0.2	14.8 ± 1.4	253.4 ± 5.4	***	***	***	***	***	***

(Data shows mean ± SEM. (Two-way ANOVA with Tukey’s multiple comparison post-hoc test) (*p ≤ 0.05, **p ≤ 0.01, ***p ≤ 0.001, ns = not significantly different).

The glia also produced chemokines, including high levels of CXCL1, monocyte chemoattractant protein 1 (MCP-1), macrophage inflammatory proteins 1α and β (MIP-1α, MIP-1β), regulated upon activation—normal T cell expressed and presumably secreted (RANTES) and low levels of eotaxin, in response to *S. aureus* challenge for OECs, TgSCs and astrocytes (Fig. 6). The levels of all these chemokines increased gradually over time from 1 to 24 h in OECs, TgSCs and astrocytes (Fig. 6). Overall, TgSCs produced higher levels of chemokines than astrocytes, which in turn secreted higher levels than OECs (with individual variations depending on chemokine and/or time-point) (Table 1). Microglia showed high levels of eotaxin as compared to OECs, TgSCs and astrocytes all throughout the time points. Eotaxin, MIP-1 β and MCP-1 all were significantly high at 1 h for microglia then decreased at 6 h and increased again at 24 h (Fig. 6).

## Discussion

Certain pathogens can enter the CNS via the nerves extending between the nasal cavity and the brain (the olfactory and trigeminal nerves)^2^. *S. aureus* is one of these microbes and can rapidly invade the brain (olfactory bulb) after injury to the nasal epithelium^12,13^. Previous studies have shown that the glia of the olfactory nerve, olfactory ensheathing cells (OECs), respond to *S. aureus* by nuclear translocation of NFκB accompanied by NO and nitrite production^12^. Capacity for intracellular survival inside OECs and/or TgSCs has previously been shown for *Streptococcus pneumoniae*^31^, *B. pseudomallei*^24^ and *Neisseria meningitidis*^32^, which can also invade the CNS via the olfactory and/or trigeminal nerves. The aim of the current study was to build on these previous findings to better understand how glia in the olfactory and trigeminal nerve, as well as astrocytes and microglia are affected by *S. aureus* infection.

We showed that *S. aureus* could adhere to and become internalized into all four glial types, over 24 h, but found differences between the capacities for intracellular survival between glia. Intracellular survival was significantly higher in astrocytes than in microglia, OECs and TgSCs, suggesting that peripheral nerve glia and microglia (a professional phagocyte) show better capacity for killing intracellular bacteria than astrocytes. Microglia internalised the *S. aureus* into the perinuclear region within 1 h of exposure to the bacteria. In contrast, OECs, TgSCs and astrocytes took longer to accumulate the bacteria in the perinuclear region. However, even at 24 h astrocytes appeared to have bacteria still within cell processes. This was reflected in the intracellular survival with astrocytes retaining significantly more viable bacteria at 24 h compared to the other cell types. The different intracellular distribution of bacteria between astrocytes and peripheral glia may hold clues to the higher capacity for intracellular survival of *S. aureus* in astrocytes^33^.

We also compared the time-course of phagocytosis between the different types of glia by exposing the cells to pHrodo-green *S. aureus* BioParticles, which become fluorescent after internalization into phagolysosomes/lysosomes, and imaging the cells over time. The results showed that microglia and astrocytes were the first cells to show significant uptake of the BioParticles in phagolysosomes/lysosomes, followed by TgSCs and then OECs. Whilst microglia, the resident macrophage of the brain^34^, were the fastest to take up the BioParticles (30 min to start their immune defence function^35^), astrocytes were faster than TgSCs or OECs. Astrocytes have previously been reported to rapidly (within 2 h) phagocytose damaged cells and synaptosomes^36,37^. Internalization of bacteria (*Streptococcus agalactiae*) by astrocytes in vitro has been shown to be slower (~ 9 h) and variable between cells^38^. Thus, it is possible that the time-course of BioParticle internalization into astrocytes may vary depending on the type of cargo (cell debris *versus* bacteria, as well as bacterial species). Previous studies have shown that OECs respond to the presence of cell debris by extending filopodia within 15 min of exposure and to internalize axonal debris within 4 h of exposure^11^. OECs and TgSCs can internalize *E. coli* bacteria 6 h post exposure^16^. The timing of these responses is relatively similar to the time-course for phagocytosis of *S. aureus* by OECs and TgSCs reported in the current study. To the best of our knowledge, the invasion frequency (percentage of attached bacteria that end up inside the cell) has not been compared between glia for any bacterial species, previously. These findings show that OECs, TgSCs, astrocytes and microglia all can respond to bacteria and bacteria-conjugated BioParticles, which are internalized into phagolysosomes within 1–4 h, with some differences between cell types regarding the percentages of cells internalizing the cargo. This time-course is similar to what has previously been reported for microglia/macrophages, which respond to bacteria and PAMPs^14^ and phagocytose bacteria-conjugated BioParticles in less than 1 h^35^ as shown in our results too.

All glia responded to *S. aureus* with secretion of multiple cytokines and chemokines. Interestingly, production of these was overall highest in microglia and TgSCs, followed by astrocytes and then OECs. This does not match the fact that *S. aureus* exhibits stronger capacity for intracellular survival in astrocytes than in OECs/TgSCs, suggesting that other inflammatory mediators than cytokines are involved in differential innate immune responses between peripheral glia and astrocytes.

We found all four glial cell types rapidly responded to *S. aureus* by secretion of TNF-α (with the highest levels being produced by TgSCs). Production of TNF-α by these cells after *S. aureus* exposure is in alignment with a previous study, in which bacterial invasion of the olfactory nerve and bulb by *S. aureus* after epithelial injury resulted in increased levels of TNF-α in primary olfactory nervous system tissue^13^. One previous study has also shown that astrocytes can produce TNF-α in response to *S. aureus*^39^*.* TNF-α is a critical component of the innate immune response against *S. aureus* brain abscess^39^, but as it is a pro-inflammatory cytokine, it can also cause damage to brain tissue and death of neurons^40^. TNF-α receptors exist both in neurons and glia, and play an important role in cell death^41^. Due to their location, OECs are often likely exposed to pathogens, and therefore mechanisms must exist that limit damage induced by pro-inflammatory cytokines. OECs and supporting cells of the primary olfactory nervous system produce pituitary adenylate cyclase activating peptide (PACAP)^42^, which protects against TNF-α-mediated death of neurons in both the olfactory nerve^43^ and brain^44^, and it has previously been suggested that PACAP may be counteracting potential harmful effects of TNF-α as a response to bacteria in the primary olfactory nervous system^13^. PACAP and its receptors are also expressed in the trigeminal nerve^45^. TNF-α expression in microglia was enhanced as early as 1 h following infection and was elevated up to 24 h which follows a similar trend as shown in a previous study^21^. We also found that the glia secreted another pro-inflammatory cytokine, IFN-γ, in response to *S. aureus*, at very high levels in microglia and at low levels for the other glial cells. In CNS injury, low levels of IFN-γ can induce neuroprotective functions by microglial cells. At high concentrations of IFN-γ, however, this neuroprotective effect decreases^46^. This was the case in microglia which produced high levels of IFN-γ and positive results for all other 23 cytokines (Supplementary Table 2).

All glia also produced regulatory cytokines IL-6 and IL-10 in response to *S. aureus*; this response was significantly delayed compared to TNF-α. Production of IL-6 by OECs^13^ and astrocytes^39^ responding to *S. aureus* has previously been shown. IL-6 plays a central role in cellular responses to nerve injury and is important for regeneration and cell survival^47,48^. IL-6 has been suggested to prevent cell death of OECs^13^ as activation of IL-6 can stimulate anti-apoptotic pathways and the IL-6 receptor is upregulated in OECs after neuronal injury^49^. *B. pseudomallei*, another bacterium that can invade the olfactory and trigeminal nerves, also stimulates production of IL-6 and TNF- α in OECs^50^. IL-10 is a potent anti-inflammatory cytokine^51,52^ that can inhibit TNF-α production, thus regulating potential damaging effects of TNF-α on tissue^53^. This is observed in microglia with high level of IL-10 production corresponding to low level of TNF-α at different time points. At 24 h post exposure to *S. aureus*, the high level of IL-10 in OECs, TgSCs, astrocytes and microglia, correlated with significantly reduced levels of TNF-α compared to different time points, perhaps suggesting that IL-10 reduced TNF-α secretion.

We found that *S. aureus* triggered production of several chemokines (chemotactic cytokines), CXCL-1, MCP-1, MIP-1α, MIP-1β and RANTES, and Eotaxin. OECs, but not TgSCs, have previously been demonstrated to respond to *E. coli* and PAMPs by CXCL-1 secretion^14^, which is demonstrated to be critical for neutrophil-dependent bacterial elimination via induction of reactive oxygen species and reactive nitrogen species^54^. Astrocytes have also been shown to secrete MIP-1, MCP-2 and MIP-1β in response to *S. aureus*^39^. These chemokines are part of the main group of cytokines attracting different populations of leukocytes, (preferentially monocytes, macrophages, eosinophils and subsets of lymphocytes) and play important roles in inflammatory responses against pathogens^55,56^. The level of MCP-1 has been shown to be increased in cerebrospinal fluid during pyogenic and tuberculous meningitis and may thus be a common responder of CNS cells to bacterial infection^57^. MCP-1 levels are also increased in plasma in meningococcal disease^58^. Microglia under non-activated conditions produce numerous cytokines such as MIP-1α, MIP-1β and MCP-1^59^ and we observed this at 24 h in the non-infected microglia.

It has previously been shown^59^ that microglia release a powerful immune response when activated. OECs and astrocytes, but not Schwann cells, mount a similar immune response to *E. coli* and PAMPs^14^. OECs and astrocytes have also been found to express higher levels of mRNA for innate immune factors than Schwann cells in a microarray study^60^. In contrast to these findings, we found that all glial cells (TgSCs, OECs, astrocytes and microglia) could all internalize and phagocytose *S. aureus* / *S. aureus* BioParticles, and that all four glial types responded to *S. aureus* with secretion of many cytokines and chemokines; in fact, microglia and TgSCs consistently secreted higher amounts of cytokines and chemokines than the other glia. In the previous study^60^, the Schwann cells were derived from the sciatic nerve and brachial plexus. Here, we used trigeminal nerve Schwann cells, which may have evolved to exhibit a more powerful immune response to pathogens than other Schwann cells, as the trigeminal nerve is likely to be more often exposed to microbes than other peripheral nerves.

Due to their unique growth-promoting properties, and because they can be relatively easy isolated from the roof of the nasal cavity, transplantation of OECs is emerging as a promising therapy for spinal cord injury repair^2,61,62^. The innate immune functions of OECs are very important in this context, due to the inflammatory environment of the spinal cord injury site (which in turn varies depending on time post-injury). Depending on the activation state of OECs, they may secrete cytokines that are pro-inflammatory or regulatory and thus have both detrimental and beneficial effects^12,14^. The fact that OECs secreted lower levels of pro-inflammatory cytokines than the other glia indicate that OECs could have immunomodulatory functions, as has been suggested previously^63^. The capacity for phagocytosis by transplanted OECs is also likely beneficial, as the cells can help clear cell debris present at the injury site. Compounds that can stimulate the phagocytic activity of OECs without causing a strong pro-inflammatory response have been suggested to potentially increase the therapeutic potential of these cells^64–68^. Thus, it is important to characterise which cytokines are expressed by OECs under various conditions.

In conclusion, these results have demonstrated that OECs, TgSCs, astrocytes and microglia can phagocytose *S. aureus.* Whilst the glia mounted an innate immune response, live bacteria could still be isolated from cells after 24 h. OECs, TgSCs (PNS) and microglia (CNS) showed stronger capacity for killing of intracellular *S. aureus* than astrocytes, however, OECs secreted the lowest amounts of both pro- and anti-inflammatory cytokines in response to bacteria, potentially suggesting an immunomodulatory function of these cells.

## Materials and methods

### Primary glia culture

Glia cultures were obtained from S100β-DsRed transgenic mice according to previously described methods^69^. Briefly, the olfactory bulb and trigeminal nerve were dissected out for preparations of OECs and TgSCs, respectively, from postnatal day seven pups. Tissue explants were plated into the wells of polystyrene 24-well plates, pre-coated with Matrigel basement membrane matrix (Corning Matrigel Basement Membrane Matrix, FAL354234). The explants were maintained in glial medium, constituting of Dulbecco's Modified Eagle Medium (DMEM), 10% foetal bovine serum (FBS) and gentamicin (Gibco 50 mg/mL), supplemented with GlutaMAX and G5 (both Gibco, added according to the manufacturer’s instructions), at 37 °C with 5% CO_2_. Cells were cultured to 80% confluency after which they were trypsinized (using Gibco TrypLE Express, 1X) and used for experiments. Primary OEC cultures were > 70% pure and TgSCs cultures were > 80% pure (based on DsRed expression) (Supplementary Fig. 1 and Supplementary Table 1).

Primary astrocytes were obtained from postnatal day three pups following a previous published protocol^70^. The brain was removed from the cranium, the olfactory bulb and cerebellum were removed. Careful removal of meninges was performed to avoid contamination of fibroblasts and meningeal cells. Forebrain was carefully separated from midbrain containing major cerebral vessels to avoid endothelial contamination. It was then cut into four smaller pieces followed by trypsinization. The cell suspension was plated in a poly-D-lysine hydrobromide (Sigma-Aldrich P6407)-coated T75 flask. After seven to eight days (90% confluency), astrocytes were separated from microglia (as overlaying microglia sit exposed) by shaking on an orbital shaker at 180 rpm for 30 min. Oligodendrocyte precursor cells were next removed by shaking the flask at 240 rpm for 6 h. The remaining astrocytes were trypsinized (using Gibco TrypLE Express) and used for experiments. Primary astrocyte cultures were > 70% pure (based on GFAP immunostaining) (Supplementary Fig. 1 and Supplementary Table 1).

Primary microglia were prepared from postnatal day 3 (P3) S100ß-DsRed transgenic mice following a previous published protocol^71^. The entire brain cell population was isolated from the brain tissue by enzymatic digestion and mechanical dissociation using Neural Tissue Dissociation Kit with GentleMACS (Miltenyi Biotec,130–093-231). The cell pellet consisting of a mixture of all brain cells was further subjected to magnetic cells sorting for microglia enrichment using CD11b/c microbeads (Miltenyi Biotec,130–093-636) according to manufacturer protocol. The different glial preparations were separately plated in plastic 24-well plates and maintained in glial medium containing Dulbecco's Modified Eagle Medium with 10% foetal bovine serum (FBS), gentamycin (Gibco, 50 mg/mL) and GlutaMAX at 37 °C with 5% CO_2_ for 5 days. Cells were replated into T-25 flasks and allowed to proliferate to 80% confluency then trypsinized (using Gibco TrypLE Express) and used for experiments. Primary microglia cultures were > 85% pure (based on Iba-1 immunostaining) (Supplementary Fig. 1 and Supplementary Table 1).

### Bacterial strain and culture conditions

*Staphylococcus aureus* (ATCC 29213) cultures were grown, from a sterile loop inoculum from a glycerol stock, in liquid Brain Heart Infusion (BHI) broth, at 37 °C in a shaking incubator (180–200 rpm) for 14–18 h^72^. After overnight incubation, the bacterial culture was centrifuged at 10,000*g* for 10 min at 20 °C. The supernatant was removed, and the bacterial pellet was washed with sterile phosphate buffered saline (PBS). The washing step was repeated twice before resuspending the bacteria in antibiotic-free medium (DMEM, 10% FBS and GlutaMAX) for experiments. Bacterial concentration was determined by plating on BHI agar plates overnight, after which the number of colony-forming units (CFU) was determined.

### In vitro infection assay

To analyse the interaction of *S. aureus* with glia, we exposed primary OEC, TgSCs, astrocytes and microglia to the bacteria. Glia were seeded in 96-well plates at 4000 cells per well and incubated at 37 °C in 5% CO_2_ until approximately 80% confluence. Monolayers were then washed and infected with *S. aureus* diluted in antibiotic-free medium at a multiplicity of infection (MOI) of 100:1^73^ or to medium alone (control) for 1 h. Adhesion of *S. aureus* to glia was determined at 1 h post exposure by washing monolayers with PBS to remove unattached bacteria, after which adherent bacteria was enumerated by CFU counts on BHI agar^74^. To determine the number of *S. aureus* CFU that have been internalised into the cells, cells were lysed using 0.05% Triton X (Sigma-Aldrich Triton X-100 laboratory grade) for 2–3 min followed by mixing with PBS and serial ten-fold dilution of the lysate. After which bacterial counts were determined on BHI agar.

To determine invasion and survival frequency of the cells, the cells were exposed to 1 h bacterial inoculation after which the cells were washed at 1 h, 6 h or 24 h with PBS and antibiotic protection media to kill extracellular bacteria. Antibiotic protection media contained penicillin 250 U/mL, streptomycin 250 U/mL (from stock Gibco Penicillin–Streptomycin, 10,000 U/mL) and gentamicin (Gibco, 50 mg/mL). Bacterial load was determined by lysing the cells and determining CFUs on BHI agar. Invasion frequency in % was determined by comparing bacterial invasion of each glial cell type between 6 h post exposure and 1 h post exposure using the following formula:

$$\% \,invasion\,frequency = \frac{{Bacterial\,load\,at\,6\,{\text{h}}\,post\,exposure}}{{Bacterial\,load\,at\,1\,{\text{h}}\,post\,exposure}} \times 100$$

Similarly, data from bacterial load at 24 h post exposure and 6 h post exposure was used to calculate intracellular survival, according to the following formula:

$$\% \,intracellular\,survival = \frac{{Bacterial \,load\,at\,24\,{\text{h}}\,post\,exposure}}{{Bacterial\,load\,at\,6\,{\text{h}}\,post\,exposure}} \times 100$$

### Phagocytosis assay

To compare the capability for phagocytosis between the different types of glia, we exposed primary OEC, TgSCs, astrocytes and microglia to pHrodo Green *S. aureus* Bioparticles Conjugate (Invitrogen) and studied internalization into lysosomes over time using live-cell imaging. Glia were seeded in 96-well plates at 4000 cells per well (in normal culture medium, DMEM, 10% FBS, gentamicin, GlutaMAX) and incubated at 37 °C in 5% CO_2_. The bioparticles were added to each well (final concentration: 10 µg/mL) from a stock solution of 1 mg/mL and live cell imaging was performed (red and green channels for DsRed cells with green bioparticles).

### Immunofluorescence

Glia seeded in 96-well plates at 4000 cells per well were fixed with 4% paraformaldehyde (PFA) for 10 min and then rinsed with PBS three times, at various time points. Further, blocking/permeabilising solution (3% bovine serum albumin in PBS with 0.3% Triton X-100) was added for 30 min at room temp on a shaker. Primary antibody, rabbit or mouse anti-*S. aureus* antibody (1:800, Abcam, ab20920) or goat anti-glial fibrillary acidic protein (GFAP) antibody (1:400, Abcam, ab53554) or rabbit ionized calcium-binding adaptor protein-1 (IBA-1) antibody (1:100, Abcam, ab178847) or Anti-LAMP2 antibody [GL2A7] (1:800, Abcam, ab13524) was added and kept at 4 °C overnight on a rocking shaker. The following day, plates were washed with PBS three times and secondary antibodies were added. These were donkey anti-rabbit IgG (highly cross-adsorbed), conjugated to Alexa Fluor 488 (1:500; Thermo Fisher Scientific, A21206), or donkey anti-goat IgG (pre-adsorbed) H&L (heavy and light chains), conjugated to Alexa Fluor 647 (1:500; Abcam, ab150135) or goat anti-rabbit IgG H&L (Alexa Fluor 594) preadsorbed (1:500; Abcam, ab150084) or donkey anti-rat IgG H&L (Alexa Fluor 647) preadsorbed (1:500, Abcam, ab150155) or goat Anti-Rat IgG H&L (Alexa Fluor 488) (1:500, ab150157) or donkey anti-goat IgG (H + L) cross-adsorbed secondary antibody, Alexa Fluor 488 (1:500; Thermo Fisher Scientific, A-11055). Cell nuclei were stained with Hoechst (1:5000, Life Technologies).

### Imaging

Lower power images were captured on a Nikon Eclipse Ti2 inverted microscope. Higher magnification images were taken using an Olympus FV3000 confocal microscope. Images were colour-balanced using Adobe Photoshop CS5 (Adobe Systems Incorporated) with the entire field of view being altered uniformly. Figures were compiled using Adobe Illustrator CS5 (Adobe Systems Incorporated). For live cell imaging, the IncuCyte Live Cell Analysis Imaging System (Sartorius) was used. Images were taken every 30 min in the red and green fluorescence channels. Analysis was performed using CellProfiler 3.0 cell image analysis software and NIS-Elements AR 5.200 software. Cell length was measured using the NIS-Elements AR 5.200 software manually, using 15–20 fields of view (FOVs), 10–15 cells per FOV, with three biological and three technical repeats.

### Cytokine assay

A set of 23 cytokines were analysed using a highly sensitive antibody-based multiplex cytokine assay kit, the Mouse Cytokine 23-Plex Group 1 kit (Bio-Rad Laboratories). Glia were seeded in 6-well plates at 100,000 cells per well (in antibiotic free culture medium) and incubated at 37 °C in 5% CO_2_. Then the in-vitro assay bacterial protocol was followed till 24 h. Cell culture supernatants were collected, filtered, and centrifuged at 1000 g for 15 min at 4 °C. The samples were stored at − 80 °C until use. On the day of experiment, samples were thawed on ice and diluted with Assay Diluent (from the kit) as directed by the manufacturer. Preparation of standards and assay techniques were followed as per the manufacturer-recommended protocol. All incubation steps were performed on a shaker and washing steps were done using a magnetic plate wash station. The beads were resuspended by shaking the plate vigorously on an orbital shaker, and immediately analysed using a Bio-Plex 200 Multiplex Reader instrument (Bio-Rad Laboratories), following the manufacturer-suggested settings. The Bio-Plex Manager Software (Bio-Rad Laboratories) was used for instrument control, data acquisition, and data analysis. The acquired data and graphs (standard curves) were exported into Microsoft Excel and then GraphPad PRISM version 7 (www.graphpad.com/scientific-software/prism) was used for further analysis and preparation of graphs for figures.

### Statistical analysis

Statistics and graphical analysis were performed using Microsoft Excel and GraphPad PRISM 7 software. One-way ANOVA with Tukey’s multiple comparison post-hoc test or Kruskal–Wallis Test with Dunn’s multiple comparison test were used for statistical analysis.

### Ethics statement

The study was carried out in compliance with the ARRIVE guidelines. All procedures were approved by Griffith University and the University Animal Ethics Committee (MSC/13/18) under the guidelines of the National Health and Medical Research Council of Australia and in accordance with the *Australian Code for the Care and Use of Animals for Scientific Purposes (8th Edition, 2013);* and in accordance with the Australian Commonwealth Office of the Gene Technology Regulator.

## Supplementary Information

Supplementary Information.
