# Supplementary material for: Antimicrobial responses of peripheral and central nervous system glia against Staphylococcus aureus
Source: Sci Rep. 2021 May 21;11:10722. doi: 10.1038/s41598-021-90252-0 (PMC8140078; doi:10.1038/s41598-021-90252-0)
Supplement: Supplementary file 1 — Supplementary Information. [file 41598_2021_90252_MOESM1_ESM.docx]

**Antimicrobial responses of peripheral and central nervous system glia against *Staphylococcus aureus***

Indra Choudhury, Anu Chacko, Ali Delbaz, Mo Chen, Souptik Basu, James St John, Flavia Huygens, Jenny Ekberg

**Supplementary data**


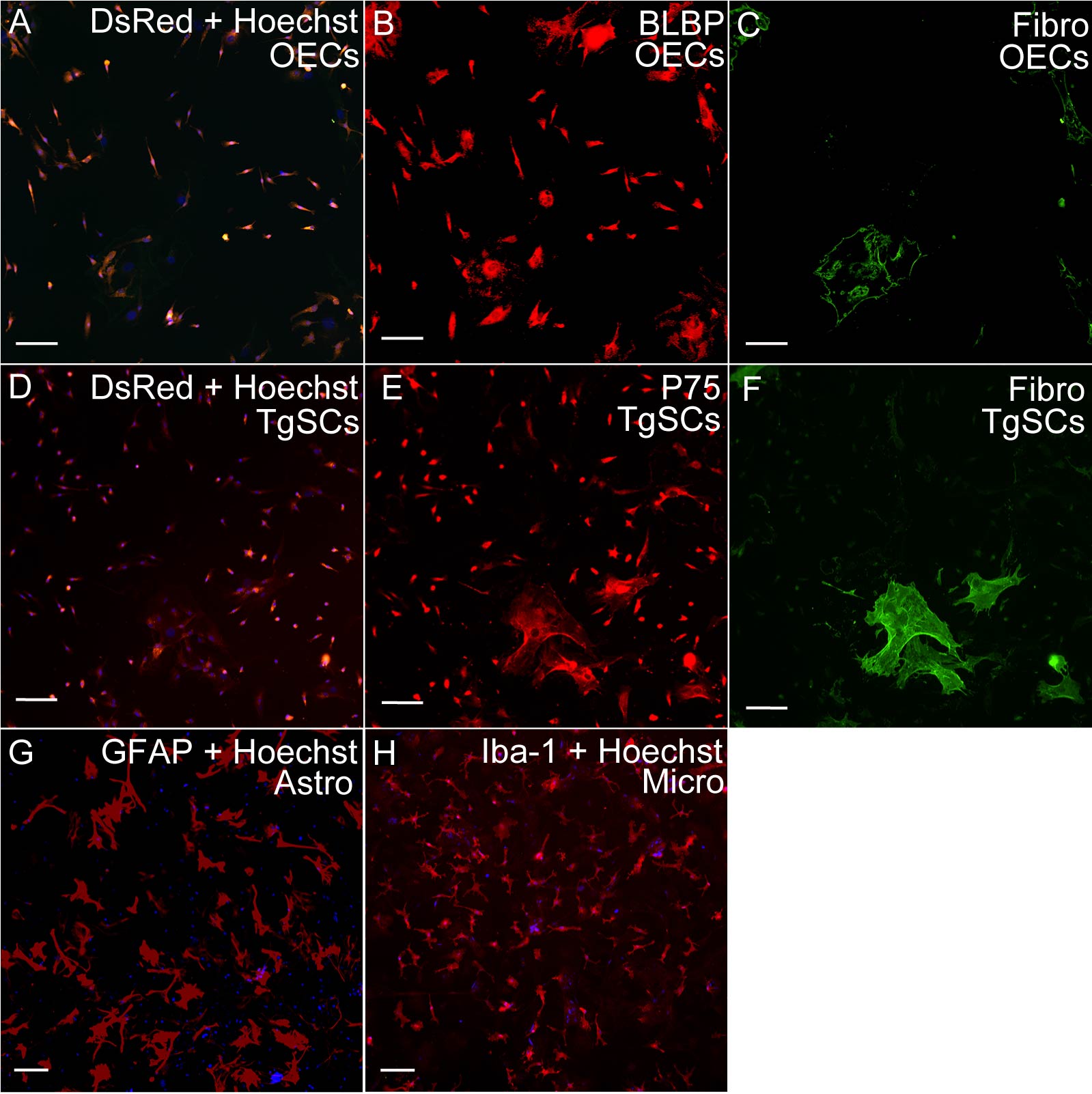


**Figure 1. Cell Purity of OECs, TgSCs, astrocytes and microglia.** Panels show primary cultures of OECs (A-C), TgSCs (D-F), astrocytes (G) and microglia (H) from S100β-DsRed mice, in which (A) OECs and (D) TgSCs express the DsRed protein. OEC cultures were also immunostained with (B) anti-BLBP and (C) anti-fibronectin. TgSCs were immunostained with (E) anti-p75ntr and (F) anti-fibronectin. (G) Astrocytes were immunostained with anti-GFAP. (H) Microglia were immunostained with anti-Iba-1. Nuclei were stained with Hoechst. Scale bar: 100 μm.

**Table 1: Percentage purity of the glial cells used in experiments.** Purity was determined by expression of DsRed or immunostaining with nuclei stain to visualise all cells.

|  | DsRed (%) | Immunostaining (%) | |
| --- | --- | --- | --- |
| OEC | 70.8 | 61.9 (anti-BLBP) | 7.5 (anti-fibronectin) |
| TgSC | 84.5 | 82.3 (anti-p75ntr) | 13.5 (anti-fibronectin) |
| Astro | - | 72.9 (anti-GFAP) |  |
| Micro | - | 87.8 (anti-Iba-1) |  |

**Table 2: Other cytokine/chemokines expressed by OECs, TgSCs, astrocytes and microglia post S. aureus exposure with comparison between the glia which were not in Fig 5 and Fig 6.**

| **Cytokine** | **Time** | **OEC (pg/mL)** | **TgSC (pg/mL)** | **Astro (pg/mL)** | **Micro (pg/mL)** |
| --- | --- | --- | --- | --- | --- |
| G-CSF | 1 h | 0 | 0 | 0 | 449.9±12.4 |
|  | 6 h | 0.1±0.1 | 104.3±7.8 | 54±1.3 | 171.8±0.2 |
|  | 24 h | 86.3±2.0 | 2388±48.5 | 632.8±9.1 | 1044.9±13.9 |
| GM-CSF | 1 h | 0 | 0 | 0 | 26.9±2 |
|  | 6 h | 0 | 7.5±1.8 | 3.4±0.9 | 65.7±1.1 |
|  | 24 h | 0 | 29.5±1.4 | 9.4±2.7 | 216.6±3.5 |
| IL-1a | 1 h | 0 | 0 | 0 | 41±0.9 |
|  | 6 h | 0 | 0 | 0 | 4.4±0.2 |
|  | 24 h | 0 | 9.6±0.1 | 0 | 6.6±0.2 |
| IL-1b | 1 h | 0 | 0 | 0 | 107.2±1.3 |
|  | 6 h | 0 | 0 | 0 | 82.4±1.5 |
|  | 24 h | 0 | 0 | 0 | 105±1.8 |
| IL-2 | 1 h | 0 | 0 | 0 | 26.5±0.9 |
|  | 6 h | 0 | 0 | 0 | 4.9±0.1 |
|  | 24 h | 0 | 0 | 0 | 6.3±0.3 |
| IL-3 | 1 h | 0 | 0 | 0 | 46.2±1.5 |
|  | 6 h | 0 | 0 | 0 | 12.2±0.4 |
|  | 24 h | 0 | 3.5±0.1 | 0 | 16.1±0.8 |
| IL-4 | 1 h | 0 | 0 | 0 | 10.7±0.2 |
|  | 6 h | 0 | 0 | 0 | 1±0.1 |
|  | 24 h | 0 | 0.5±0.1 | 0 | 1.2±0.1 |
| IL-5 | 1 h | 0 | 0 | 0 | 23±0.5 |
|  | 6 h | 0 | 1.5±0.1 | 0 | 1.9±0.1 |
|  | 24 h | 0 | 3.9±0.1 | 1.7±0.5 | 4.1±0.3 |
| IL-9 | 1 h | 0 | 0 | 0 | 303.4±3.6 |
|  | 6 h | 0 | 5.5±1 | 0.9 | 212.4±3.2 |
|  | 24 h | 2.3±0.4 | 14.9±1.5 | 7±0.6 | 300.7±6.5 |
| IL-12 (p-40) | 1 h | 38.9 | 74.6±5.7 | 0 | 1357.4±14.5 |
|  | 6 h | 97±25.9 | 80.8±33.8 | 147.8±16.8 | 993±33.2 |
|  | 24 h | 251.2±2.7 | 1553.2±20.4 | 459.2±36.9 | 1199.7±23.6 |
| IL-12 (p-70) | 1 h | 8.9±4.5 | 10.2±1.1 | 2.2±0.8 | 446.7±15.1 |
|  | 6 h | 21.8±2.4 | 46.4±1.7 | 28.7±1.8 | 77.1±5.4 |
|  | 24 h | 29.2±3.2 | 92.8 | 39.1±1.3 | 137.5±4.7 |
| IL-13 | 1 h | 0 | 0 | 0 | 24.3±1.7 |
|  | 6 h | 0 | 0 | 0 | 2.3±1.1 |
|  | 24 h | 0 | 0 | 0 | 12.7±1.6 |
| IL-17 | 1 h | 0.3±0.2 | 0 | 0 | 18.5±0.2 |
|  | 6 h | 0 | 1.1±0.1 | 0.1 | 3.5±0.2 |
|  | 24 h | 0.3±0.1 | 3.4±0.2 | 0.5 | 4.4±0.2 |
